# Prognostic value of blood inflammatory composite markers in the survival of pediatric patients with secondary hemophagocytic lymphohistiocytosis

**DOI:** 10.3389/fped.2025.1458490

**Published:** 2025-07-24

**Authors:** Nandu Luo, Xin Xie, Yan Chen, Zuochen Du, Pei Huang

**Affiliations:** ^1^ Department of Pediatrics, Affiliated Hospital of Zunyi Medical University, Zunyi, China; ^2^ Guizhou Children’s Hospital, Zunyi, China; ^3^ Collaborative Innovation Center for Tissue Injury Repair and Regenerative Medicine of Zunyi Medical University, Zunyi, China

**Keywords:** hemophagocytic lymphohistiocytosis, prognostic factors, LAR, RPR, children

## Abstract

**Objective:**

This study aimed to explore the prognostic value of blood inflammatory composite markers in the survival of pediatric patients diagnosed with secondary hemophagocytic lymphohistiocytosis (sHLH).

**Methods:**

Clinical data from 138 newly diagnosed sHLH patients hospitalized between January 2012 and October 2023 were analyzed. Receiver operating characteristic curve analysis was used to determine cutoff values and evaluate predictive accuracy, while Cox regression analysis was employed to identify prognostic factors.

**Results:**

The median age of the 138 sHLH patients was 38 months, with a female-to-male ratio of 0.92. Infection was identified as the most common cause of sHLH, 52.9% testing positive for the epstein-barr virus (EBV). Clinical features included decreased blood cell counts in 87.0% of patients, hypofibrinogenemia in 55.07%, hypertriglyceridemia in 46.38%, and elevated ferritin levels in 94.2%. Additionally, all patients experienced fever, while hepatomegaly and splenomegaly were observed in 66.67% and 76.81%, respectively. During the study, 48 patients died. Cox regression analysis identified red blood cell distribution width (RDW) ≥14.35%, fibrinogen <1.5 g/L, red blood cell distribution width to platelet ratio (RPR) ≥0.36, and lactate dehydrogenase to serum albumin ratio (LAR) ≥56.02 as significant predictors of decreased survival.

**Conclusion:**

This study provides preliminary evidence that accessible inflammatory markers like LAR and RPR may assist in early prognostic assessment of pediatric sHLH. These findings highlight the potential utility of routine blood parameters, warranting further validation in larger, stratified cohorts.

## 1 Introduction

Hemophagocytic lymphohistiocytosis (HLH) is a subtype of histiocytosis characterized by dysfunction of cytotoxic T cells (CTLs) and natural killer (NK) cells, leading to impaired antigen clearance and excessive activation of the monocyte-macrophage system. This dysregulation results in cytokine overproduction and clinical manifestations such as fever, hepatosplenomegaly, pancytopenia, hypertriglyceridemia, hypofibrinogenemia, hyperferritinemia, and hemophagocytosis in bone marrow tissue (1, 2). Secondary HLH (sHLH) is the most common form and has diverse etiologies, including malignancies, rheumatic and immune diseases, and infections (1–3). Due to immune dysfunction, patients with HLH experience hypercytokinemia, which causes ongoing tissue damage and initiates a cascade of inflammatory events. This cascade contributes to the disease's rapid progression and high mortality (3). Without timely intervention, the majority of patients with HLH die from severe infections, visceral bleeding, neutropenia or multiple organ failure, with a median survival time of less than two months (4, 5). Even with treatment, approximately one-third of patients with HLH still die, with more than half of these deaths occurring in the early stages (within 30 days) of the disease (6). Consequently, identifying prognostic indicators for HLH are of great significance for early identification of critically ill patients and improving survival outcomes.

Inflammatory mechanisms are central to HLH-associated organ dysfunction and mortality. Patients often exhibit elevated levels of cytokines (IL-6, IL-10, IFN-*γ*, TNF-α) and serological markers (ALT, AST, CRP, ferritin, LDH, TG), which are indicative of disease severity and poor prognosis. However, limited access to inflammatory markers, such as cytokines, ferritin, and sCD25, in certain regions may hinder effective risk stratification and delay timely clinical decision-making, potentially contributing to poorer patient outcomes. Therefore, identifying novel, widely accessible biomarkers are critical for improving the evaluation and management of HLH prognosis (7, 8).

Previous studies have confirmed that blood inflammatory composite markers can effectively predict prognosis in inflammatory diseases and play an important role in risk stratification. For example, markers like the neutrophil-to-lymphocyte ratio (NLR) have demonstrated utility in predicting 90-day mortality in septic patients, with an AUC of 0.66 (sensitivity = 69.57%, specificity = 61.44%) (9). Albumin bilirubin score (ALBI) has shown an AUC of 0.693 in predicting 28-day mortality in non-Hodgkin lymphoma-associated HLH (10), while prognostic nutritional index (PNI) has demonstrated an AUC of 0.64 (95% CI: 0.61–0.67) for severe sepsis and 0.69 (95% CI: 0.60–0.78) for septic shock (11). Other markers include the red blood cell distribution width-to-platelet ratio (RPR) (12), C-reactive protein-to-albumin ratio (CAR) (13), lactate dehydrogenase-to-albumin ratio (LAR) (14), alanine aminotransferase-to-aspartate aminotransferase ratio (De Ritis) (15), platelet-to-lymphocyte ratio (PLR) (9), all of which have been associated with prognosis in various conditions.

Despite growing recognition of the prognostic value of blood inflammatory composite markers in various inflammatory diseases, most existing studies have predominantly focused on adult populations. However, pediatric sHLH differs significantly from adult-onset cases in several aspects, including immune system maturity, underlying etiologies (with a higher prevalence of infection-related triggers such as EBV in children), clinical presentation, and treatment responses (16). These distinctions highlight the need for pediatric-specific prognostic research. Currently, there is a notable lack of studies examining the prognostic relevance of blood inflammatory composite markers in children with sHLH. These markers, which are inexpensive and routinely available in clinical practice, have shown promise in adult inflammatory conditions but remain underexplored in pediatric HLH. To address this gap, the present study serves as a proof-of-concept evaluation to explore whether routinely available composite inflammatory markers can provide preliminary prognostic insights in an undifferentiated pediatric sHLH cohort, aiming to offer an initial reference framework for early risk stratification using accessible laboratory parameters.

## 2 Materials and methods

### 2.1 Patients

This retrospective cohort study included patients diagnosed with HLH at the Department of Pediatrics, Affiliated Hospital of Zunyi Medical University from January 2012 to October 2023. All patients met the HLH-2004 diagnostic criteria (17). Exclusion criteria included primary HLH with HLH-related pathogenic gene defects, patients older than 14 years, patients with incomplete clinical data, and patients diagnosed and/or treated at other facilities. The patients in this study received treatments tailored to their clinical presentation, which including: The HLH-94/HLH-2004 protocols. Blood purification treatments, including plasma exchange or continuous renal replacement therapy (CRRT) for managing severe inflammation and organ failure. Blood transfusion therapy to address cytopenias and maintain adequate hemoglobin levels. Human immunoglobulin infusion (IVIG) for immunomodulation and infection control. Anti-infection and antiviral therapy targeting underlying triggers such as bacterial or viral infections (e.g., EBV). This study was conducted at the Affiliated Hospital of Zunyi Medical University and was approved by the Ethics Committee (approval number: KLL-2023–599) with waiver of informed consent requirement since this is a retrospective study.

### 2.2 Variables and outcomes

Clinical data of all eligible sHLH patients were collected, including demographic information (gender, age), etiology, hepatosplenomegaly, and laboratory indicators (blood routine, liver function, coagulation function, blood lipids, C-reactive protein and serum ferritin) at the time of diagnosis. The peripheral blood inflammation parameters analyzed in this study included NLR, PLR, RPR, ALBI, De Ritis, LAR, CAR, and PNI. These indices were calculated based on in-hospital blood indicators such as complete blood count, biochemistry, coagulation function, lipids, ferritin, and CRP. Follow-up extended from diagnosis to the patient death or October 30, 2023, via outpatient or telephone contact, with overall survival (OS) time (days) defined as the duration from diagnosis to various time points: 1 week, 2 weeks, 4 weeks, two months, three months, and the last follow-up, or death for any reason.

### 2.3 Statistical methods

Data analysis was performed using SPSS (v29.0), GraphPad Prism (v8.0). Measurement data were presented as mean ± standard deviation (x̅ ± s), median and interquartile ranges [M (Q1, Q3)], depending on distribution. Categorical data were presented as *n* (%). Normally distributed data were compared using the unpaired t-test, while non-normally distributed data were analyzed using the Mann–Whitney U test. Categorical variables were compared using the chi-squared test. ANC, HB, PLT, FIB, and TC were categorized based on the threshold values defined in the HLH diagnostic criteria. AST, and ALT were categorized as elevated if they were ≥2 times the upper limit of the reference values at our institution. Elevated LDH level was defined as >1,000 IU/L (18, 19). Hypoalbuminemia was defined as albumin ≤30 g/L. For NLR, PLR, RPR, RDW, LAR, CAR, ALBI, D-dimer, PNI, and SF, the cutoff values were determined using ROC curve analysis. Cox regression was employed to identify prognostic factors, and the Kaplan–Meier method was applied to plot survival curves for different subgroups. Statistical significance was defined as *P* < 0.05 for two-sided tests.

## 3 Results

### 3.1 General information

**Figure 1:**
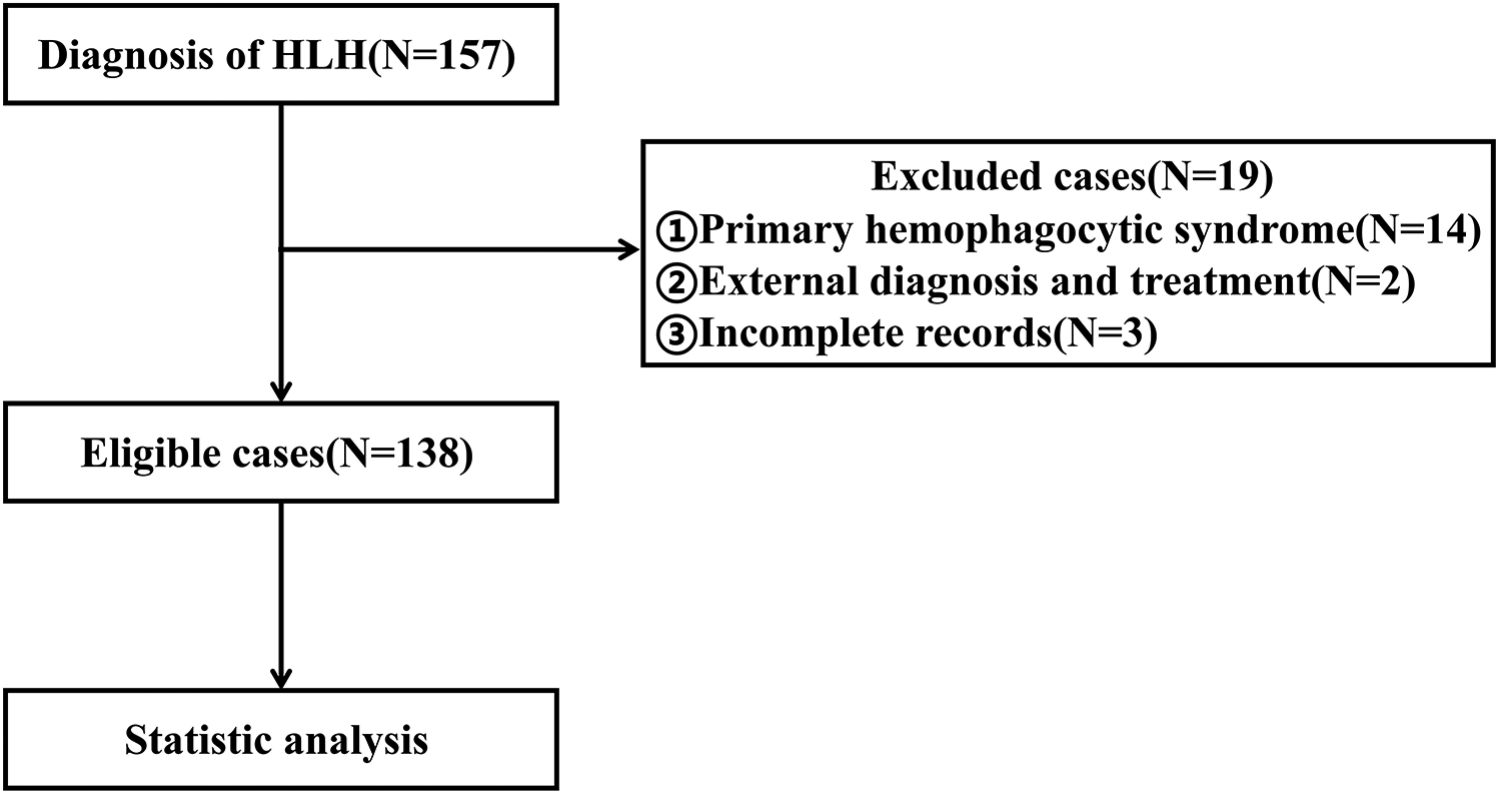
Flowchart illustrating the patient selection process.

Between January 2012 and October 2023, a total of 157 patients diagnosed with HLH received treatment at our hospital. After applying strict screening criteria, 138 patients were included in this study (Figure 1). The cohort consisted of 72 males (52.17%) and 66 females (47.83%), with a median age of 38 months. Among the cases, 99 (71.74%) were attributed to infection, with 73 cases (52.90%) specifically linked to EB virus. Thirteen cases (9.42%) were associated with tumors or autoimmune diseases, while the etiology was unknown in 26 cases (18.84%). By the follow-up deadline (median follow up: 18.62 months), 48 patients (34.79%) had died. The causes of death included 36 cases of multiple organ dysfunction syndrome (MODS), 6 cases of respiratory failure, 5 cases of circulatory failure, and 1 case of intracranial hemorrhage.

Laboratory tests revealed a median white blood cell count (WBC) of 2.17 × 10^9^/L. Among the cases, 87 (63.04%) had an absolute neutrophil count (ANC) below 1 × 10^9^/L, 62 (44.93%) exhibited hemoglobin level (HB) below 90 g/L, and 109 (78.99%) had platelet counts (PLT) below 100 × 10^9^/L. Additionally, 76 cases (55.07%) had fibrinogen levels below 1.5 g/L, 64 cases (46.38%) presented triglycerides above 3 mmol/L, 74 cases (53.62%) had alanine aminotransferase (ALT) levels ≥80 U/L, and 105 cases (76.09%) exhibited aspartate aminotransferase (AST) levels ≥100 U/L. Furthermore, 70 cases (50.72%) had albumin levels ≤ 30 g/L. Median levels for C-reactive protein (CRP), red cell distribution width (RDW), and serum ferritin were 17.69 mg/L, 14.20%, and 4,125.95 µg/L, respectively. Regarding clinical manifestations, all 138 cases (100%) experienced fever, 92 cases (66.67%) presented hepatomegaly, and 106 cases (76.81%) exhibited splenomegaly (Table 1).

**Table 1:** Clinical characteristics of 138 patients with sHLH.

Characteristics	Num	Percentage (%)
Age (month, median, range)	38 (14.75, 87)	
≤2 years	55	39.86%
>2 years	83	60.14%
Sex		
Male	72	52.17%
Female	66	47.83%
Pathogenesis		
Infection-associated	99	71.74%
Tumor/Autoimmune-associated	13	9.42%
Unknown reason	26	18.84%
EBV virus infection		
Positive	73	52.90%
Negative	65	47.10%
WBC (×10^9^/L) (median, range)	2.17 (1.38, 4.01)	
ANC (×10^9^/L) (median, range)	0.79 (0.35, 1.68)	
<1 × 10^9^/L	87	63.04%
≥1 × 10^9^/L	51	36.96%
HB (g/L) ($\bar{x}$±s)	92.01 ± 18.80	
<90 g/L	62	44.93%
≥90 g/L	76	55.07%
PLT (×10^9^/L) (median, range)	58.5 (35.0, 88.75)	
<100 × 10^9^/L	109	78.99%
≥100 × 10^9^/L	29	21.01%
FIB(g/L) (median, range)	1.40 (0.82, 1.87)	
<1.5 g/L	76	55.07%
≥1.5 g/L	62	44.93%
Triglycerides (median, range)	3.32 (2.33, 4.24)	
≤3 mmol/L	74	53.62%
>3 mmol/L	64	46.38%
ALT (U/L) (median, range)	116.5 (46.5, 327.0)	
≥80 U/L	83	60.14%
<80 U/L	55	39.86%
AST (U/L) (median, range)	237.0 (102.25, 719.25)	
≥100 U/L	105	76.09%
Characteristics	Num	Percentage (%)
<100 U/L	33	23.91%
CRP (mg/L) (median, range)	17.69 (5.37, 63.15)	
Total bilirubin (median, range)	12.50 (7.78, 44.85)	
Albumin ($\bar{x}$ ± s)	30.22 ± 5.51	
≤30 g/L	70	50.72%
>30 g/L	68	49.28%
RDW(%) (median, range)	14.20 (13.40, 15.83)	
LDH (U/L) (median, range)	1,050.5 (699.0, 1,990.0)	
≥1,000 U/L	73	52.90%
<1,000 U/L	65	47.10%
Ferritin (μg/L) (median, range)	4,125.95 (1,500.0,13, 801.50)	
Fever	138	100.00%
Hepatomegaly	92	66.67%
Splenomegaly	106	76.81%

WBC, white blood cell; ANC, absolut eneutrophil count; HB, hemoglobin; PLT, platelet; FIB, fibrinogen; ALT, alanine aminotransferase; AST, aspartate aminotransferase; CRP, C-reactive protein; LDH, lactic dehydrogenase. RDW, red blood cell distribution width.

### 3.2 Comparison of peripheral blood cell parameters in different prognoses

**Figure 2:**
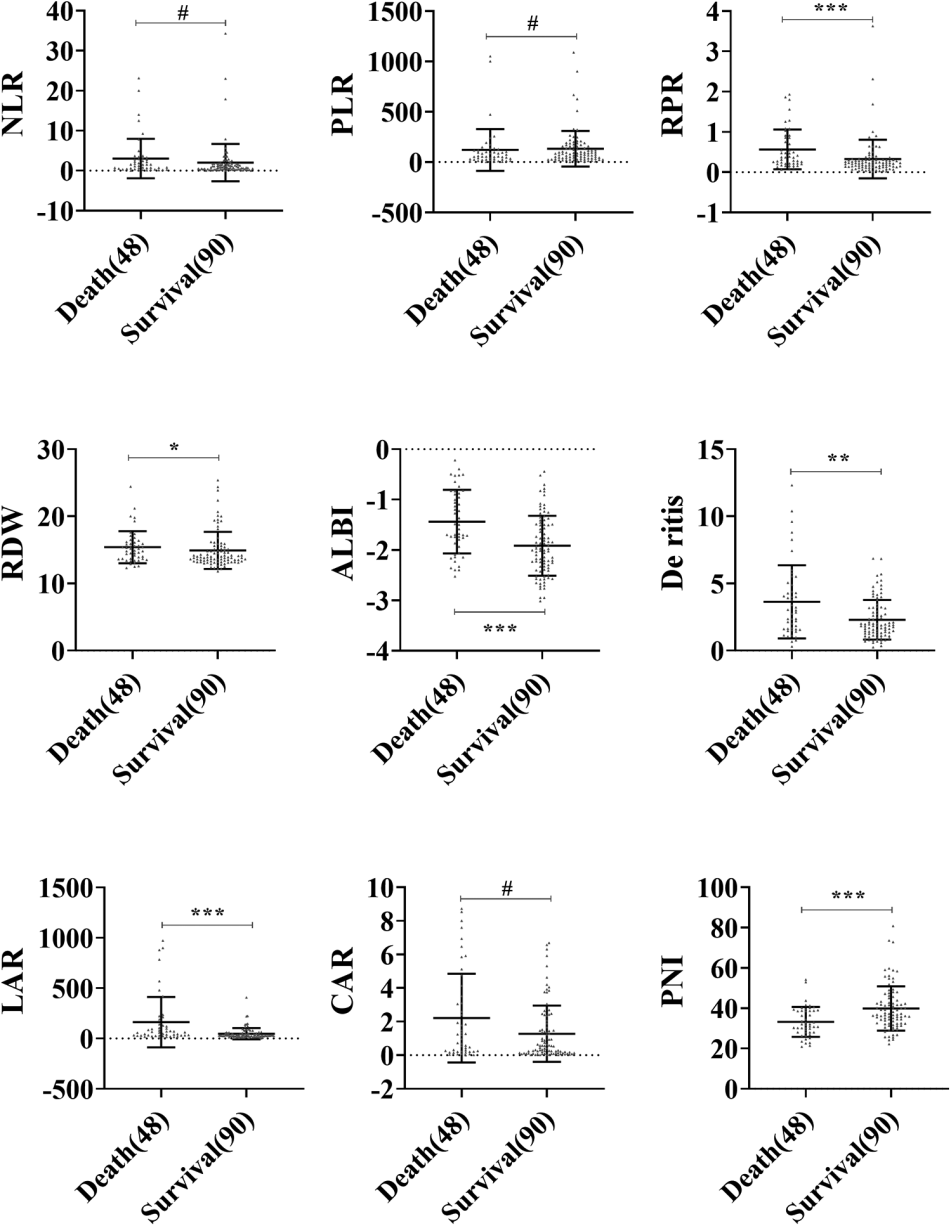
Comparison of blood inflammatory composite markers between the survival and death groups in sHLH. Statistical significance is indicated as follows: #: no statistical significance, * *P* < 0.05, ** *P* < 0.01, *** *P* < 0.001.

A comparison of peripheral blood inflammatory parameters in sHLH showed that the levels of RPR, RDW, ALBI, De Ritis, and LAR were significantly higher in the death group compared to the survival group (*P* < 0.05). Additionally, PNI was notably lower in the death group compared to the survival group (*P* < 0.001). However, no statistically significant differences (*P* > 0.05) were observed for NLR, PLR, and CAR between the two groups (Figure 2).

### 3.3 Predictive value of inflammation indices in pediatrics sHLH

**Figure 3:**
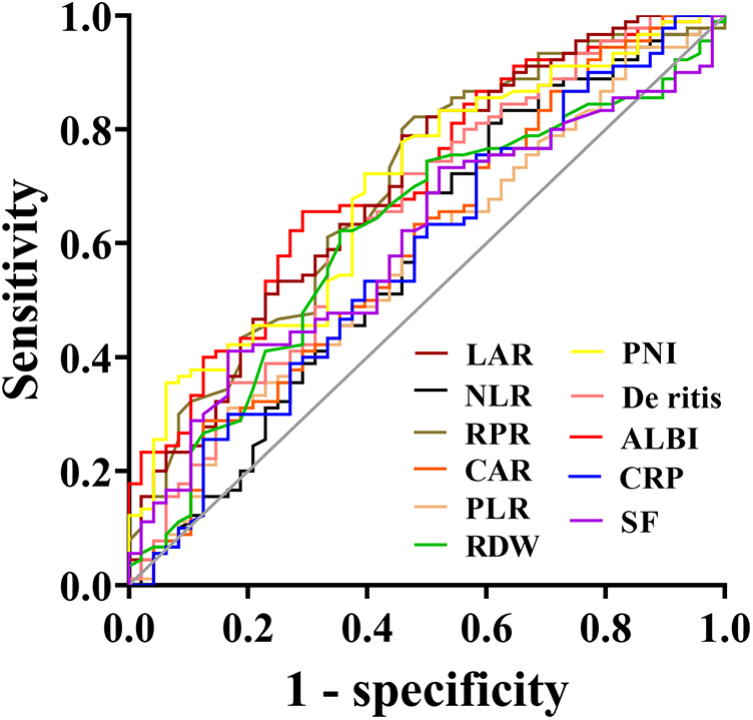
Receiver operating characteristic (ROC) curves of blood inflammatory composite markers predicting the prognosis of sHLH.

The predictive value of peripheral blood inflammation parameters for the prognosis of sHLH patients showed that all indicators had an AUC greater than 0.500. Among these, ALBI exhibited the highest AUC of 0.710, with an optimal cut-off value of −1.76 (sensitivity = 70.6%, specificity = 65.6%). LAR followed with an AUC of 0.697 and an optimal cut-off value of 56.02 (sensitivity = 54.2%, specificity = 78.9%). CAR demonstrated the highest sensitivity (87.5%) at an optimal cut-off value of 0.15, while SF showed a sensitivity of 83.3% at an optimal cut-off value of 1,897 μg/L. NLR demonstrated the highest specificity of (83.3%) with the optimal cut-off value of 2.19, followed by RPR with a specificity of 82.2% at a cut-off value of 0.36 (Table 2 and Figure 3).

**Table 2:** Receiver operating characteristic (ROC) curves for determining the optimal cut-off value.

Characteristic	AUC (95%CI)	Sensitivity	Specificity	Youden index	Cutoff value
NLR	0.582 (0.477–0.686)	0.375	0.833	0.208	2.19
PLR	0.573 (0.473–0.674)	0.542	0.622	0.164	62.33
RPR	0.695 (0.603–0.788)	0.542	0.822	0.364	0.36
RDW	0.616 (0.518–0.714)	0.646	0.622	0.268	14.35
LAR	0.697 (0.604–0.790)	0.542	0.789	0.331	56.02
CAR	0.595 (0.493–0.697)	0.875	0.289	0.164	0.15
CRP	0.579 (0.477–0.681)	0.417	0.756	0.173	48.53
ALBI	0.710 (0.621–0.799)	0.708	0.656	0.364	−1.76
De ritis	0.652 (0.553–0.750)	0.646	0.633	0.279	2.13
PNI	0.693 (0.602–0.784)	0.854	0.511	0.326	33.53
SF	0.601 (0.505–0.697)	0.833	0.411	0.244	1,897

AUC, area under curve; CI, confidence interval.

### 3.4 Survival analysis

**Figure 4:**
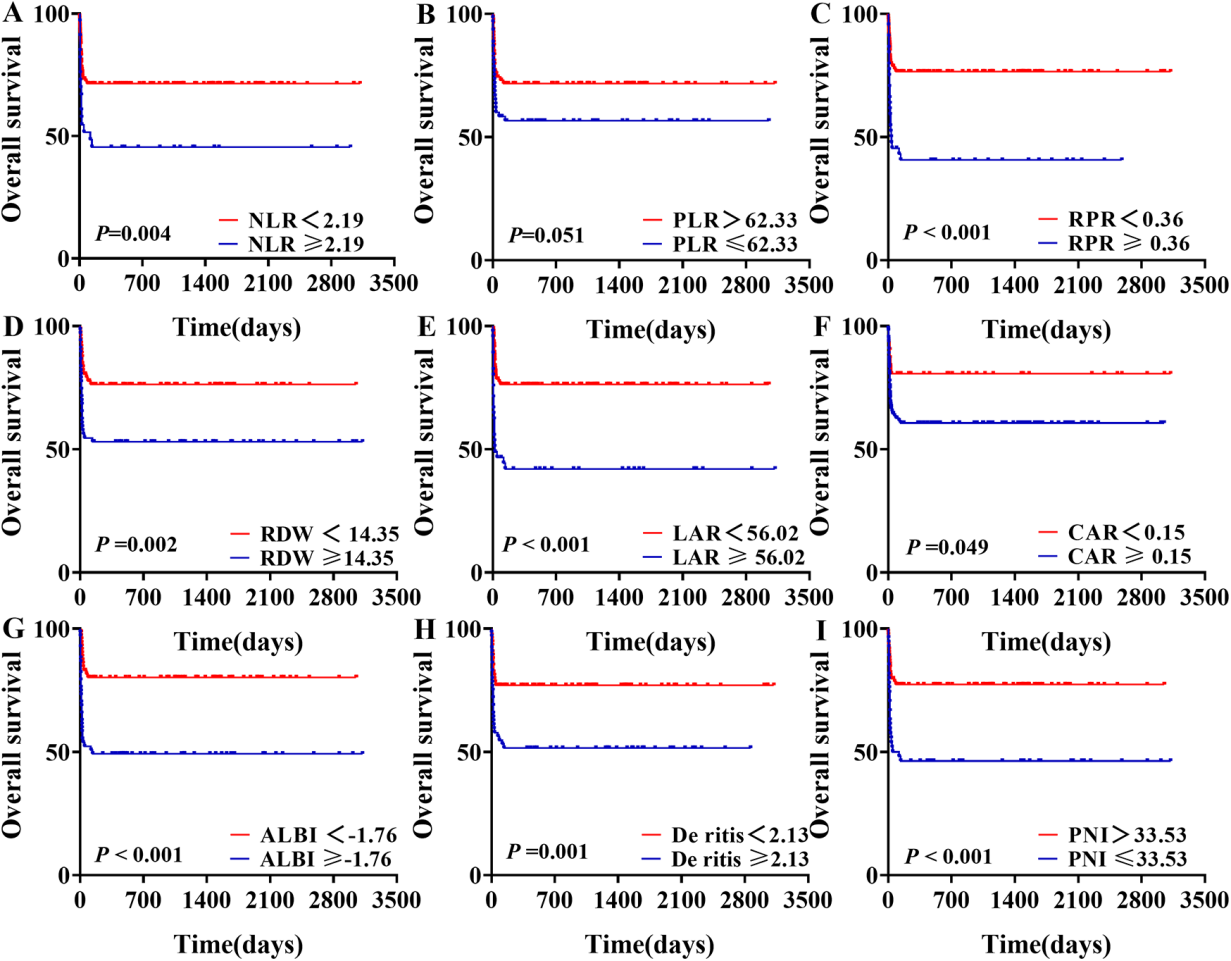
Survival analysis of sHLH patients stratified by different levels of blood inflammatory composite markers.

Survival analysis was performed for groups stratified based on the optimal cutoff values of various inflammation indexes. Patients with NLR ≥ 2.19, RPR ≥ 0.36, RDW ≥ 14.35, LAR ≥ 56.02, CAR ≥ 0.15, ALBI ≥ −1.76, De Ritis ≥ 2.13, and PNI ≤ 33.53 exhibited significantly worse survival outcomes compared to their respective control group (*P* < 0.05). However, there was no statistically significant difference in survival outcomes between the PLR ≤ 62.33 group and the PLR > 62.33 group (*P* = 0.051). From the survival plots, it was evident that mortality in our study predominantly occurred within the first two months (Figure 4).

### 3.5 Prognostic analysis of sHLH in children

Univariate regression analysis revealed that age, PLT, HB, RDW, ALT, AST, albumin, LDH, FIB, SF, CRP, NLR, RPR, LAR, ALBI, De Ritis, and PNI were associated with patient prognosis. Multivariate Cox regression analysis showed that, after adjusting for factors such as age, gender, and etiology, a higher LAR was associated with an increased risk of mortality (HR = 2.884, 95% CI = 1.588–5.237). Similarly, RPR was identified as a significant risk factor for mortality (HR = 2.136, 95% CI = 1.182–3.861). Additionally, high RDW levels (HR = 1.968) and low FIB levels (HR = 2.258) were also determined to be significant risk factors for mortality in sHLH patients (*P* < 0.05) (Table 3).

**Table 3:** Prognostic factors of overall survival of 138 patients with sHLH.

Characteristic (Ref)	Univariate Cox analysis		Multivariate Cox analysis	
	HR	*P*-value	HR	*P*-value
Age (≥ 2years)	1.931 (1.094–3.409)	0.023		
Gender (Female)	1.030 (0.584–1.817)	0.919		
EBV infection (No)	0.944 (0.536–1.664)	0.843		
Hepatomegaly (Yes)	0.627 (0.353–1.113)	0.111		
Splenomegaly (No)	1.927 (0.865–4.297)	0.109		
ANC (<1 × 10^9^/L)	0.819 (0.459–1.462)	0.500		
PLT (≥100 × 10^9^/L)	2.628 (1.041–6.638)	0.041		
HB (≥90 g/L)	2.492 (1.388–4.474)	0.002		
RDW (<14.35%)	2.466 (1.364–4.460)	0.003	1.968 (1.059–3.658)	0.032
Triglycerides (≤3 mmol/L)	1.316 (0.741–2.337)	0.348		
ALT (<80 U/L)	1.902 (1.020–3.546)	0.043		
AST (<100 U/L)	2.687 (1.142–6.324)	0.024		
Albumin (>30 g/L)	3.020 (1.619–5.635)	0.001		
LDH (<1,000 U/L)	2.193 (1.203–3.998)	0.010		
Fib (≥1.5 g/L)	4.024 (2.003–8.084)	<0.001	2.258 (1.082–4.713)	0.030
SF (<1,897 μg/L)	2.810 (1.314–6.007)	0.008		
CRP (<48.53)	1.924 (1.083–3.418)	0.026		
NLR (<2.19)	2.305 (1.284–4.140)	0.005		
PLR (>62.33)	1.746 (0.990–3.082)	0.054		
RPR (<0.36)	3.280 (1.857–5.792)	<0.001	2.136 (1.182–3.861)	0.012
CAR (<0.15)	2.303 (0.979–5.418)	0.056		
LAR (<56.02)	3.555 (2.011–6.284)	<0.001	2.884 (1.588–5.237)	0.001
ALBI (<−1.76)	3.477 (1.863–6.488)	<0.001		
De ritis (<2.13)	2.569 (1.421–4.644)	0.002		
PNI (>33.53)	2.898 (1.623–5.177)	<0.001		

### 3.6 Hazard ratios of LAR and RPR index for overall survival in subgroups

Subgroup analysis was conducted to access the impact of confounding factors on the prognosis of sHLH patients, including age, gender, EBV infection, absolute neutrophil count, hemoglobin, platelets, fibrinogen, lactate dehydrogenase, triglycerides, serum ferritin, hepatomegaly, and splenomegaly. LAR and RPR demonstrated significant predictive value across multiple variable subgroups. Interestingly, in the subgroup analysis, RPR showed an interaction within the age subgroups, LAR showed an interaction within the gender subgroups. However, for the remaining subgroups, there were no statistically significant differences in the predictive value of LAR or RPR when combined with baseline characteristics for predicting mortality risk in children with sHLH. These findings suggest that both LAR and RPR are potentialprognostic indicators regardless of most baseline characteristics (Tables 4, 5).

**Table 4:** Hazard ratios of RPR index for overall survival according to subgroups.

Subgroup	Count	Percent	HR (95% CI)	*P*-value	*P*-interaction
EBV					0.739
No	65	47.1	3.76 (1.64–8.61)	0.002	
Yes	73	52.9	3.22 (1.44–7.18)	0.004	
Sex					0.351
Female	66	47.8	4.25 (1.78–10.18)	0.001	
Male	72	52.2	2.68 (1.23–5.86)	0.013	
Age					0.042
>2y	83	60.1	5.82 (2.43–13.94)	<0.001	
≤2y	55	39.9	1.85 (0.85–4.03)	0.122	
ANC					0.169
<1 × 10^9^/L	62	44.9	2.35 (1.07–5.20)	0.034	
≥1 × 10^9^/L	76	55.1	5.63 (2.29–13.87)	<0.001	
HB					0.981
≥90 g/L	87	63	3.28 (1.51–7.11)	0.003	
<90 g/L	51	37	2.91 (1.24–6.85)	0.014	
PLT					0.325
≥100 × 10^9^/L	42	30.4	7.66 (1.94–30.26)	0.004	
<100 × 10^9^/L	96	69.6	3.27 (1.65–6.51)	0.001	
TG					0.235
≤3 mmol/L	64	46.4	5.29 (2.10–13.33)	<0.001	
>3 mmol/L	74	53.6	2.43 (1.15–5.15)	0.02	
LDH					0.644
<1,000 U/L	65	47.1	2.70 (1.01–7.27)	0.049	
≥1,000 U/L	73	52.9	3.28 (1.62–6.63)	0.001	
FIB					0.634
≥1.5 g/L	62	44.9	3.52 (0.99–12.47)	0.052	
<1.5 g/L	76	55.1	2.39 (1.26–4.55)	0.008	
SF					0.931
<1,897 ug/L	45	32.6	2.97 (0.71–12.46)	0.136	
≥1,897 ug/L	93	67.4	2.92 (1.56–5.46)	0.001	
Hepatomegaly					0.700
No	46	33.3	2.92 (1.20–7.08)	0.018	
Yes	92	66.7	3.78 (1.78–8.00)	0.001	
Splenomegaly					0.102
No	32	23.2	9.03 (1.94–42.06)	0.005	
Yes	106	76.8	2.63 (1.42–4.87)	0.002	

**Table 5:** Hazard ratios of LAR index for overall survival according to subgroups.

Subgroup	Count	Percent	HR (95% CI)	*P*-value	*P*-interaction
EBV					0.145
No	65	47.1	2.36 (1.02–5.47)	0.045	
Yes	73	52.9	5.57 (2.40–12.96)	<0.001	
Sex					0.021
Female	66	47.8	7.31 (3.04–17.59)	<0.001	
Male	72	52.2	1.99 (0.92–4.31)	0.08	
Age					0.448
>2 years	83	60.1	4.20 (1.81–9.74)	0.001	
≤2 years	55	39.9	2.83 (1.30–6.13)	0.008	
ANC					0.757
<1 × 10^9^/L	62	44.9	3.13 (1.43–6.82)	0.004	
≥1 × 10^9^/L	76	55.1	3.73 (1.61–8.63)	0.002	
HB					0.701
≥90 g/L	87	63	3.22 (1.49–6.98)	0.003	
<90 g/L	51	37	3.72 (1.56–8.91)	0.003	
PLT					0.627
≥100 × 10^9^/L	42	30.4	2.85 (0.93–8.75)	0.067	
<100 × 10^9^/L	96	69.6	3.86 (1.96–7.61)	<0.001	
TG					0.548
≤3 mmol/L	64	46.4	2.95 (1.20–7.23)	0.018	
>3 mmol/L	74	53.6	4.02 (1.85–8.75)	<0.001	
FIB					0.434
≥1.5 g/L	62	44.9	1.54 (0.33–7.24)	0.587	
<1.5 g/L	76	55.1	2.96 (1.52–5.73)	0.001	
SF					0.349
<1,897 ug/L	45	32.6	5.61 (1.12–28.03)	0.036	
≥1,897 ug/L	93	67.4	2.70 (1.43–5.10)	0.002	
Hepatomegaly					0.822
No	46	33.3	3.79 (1.54–9.32)	0.004	
Yes	92	66.7	3.30 (1.57–6.92)	0.002	
Splenomegaly					0.548
No	32	23.2	5.06 (1.12–22.85)	0.035	
Yes	106	76.8	3.16 (1.70–5.86)	<0.001	

### 3.7 RPR combined with LAR exhibited better predictive value

**Figure 5:**
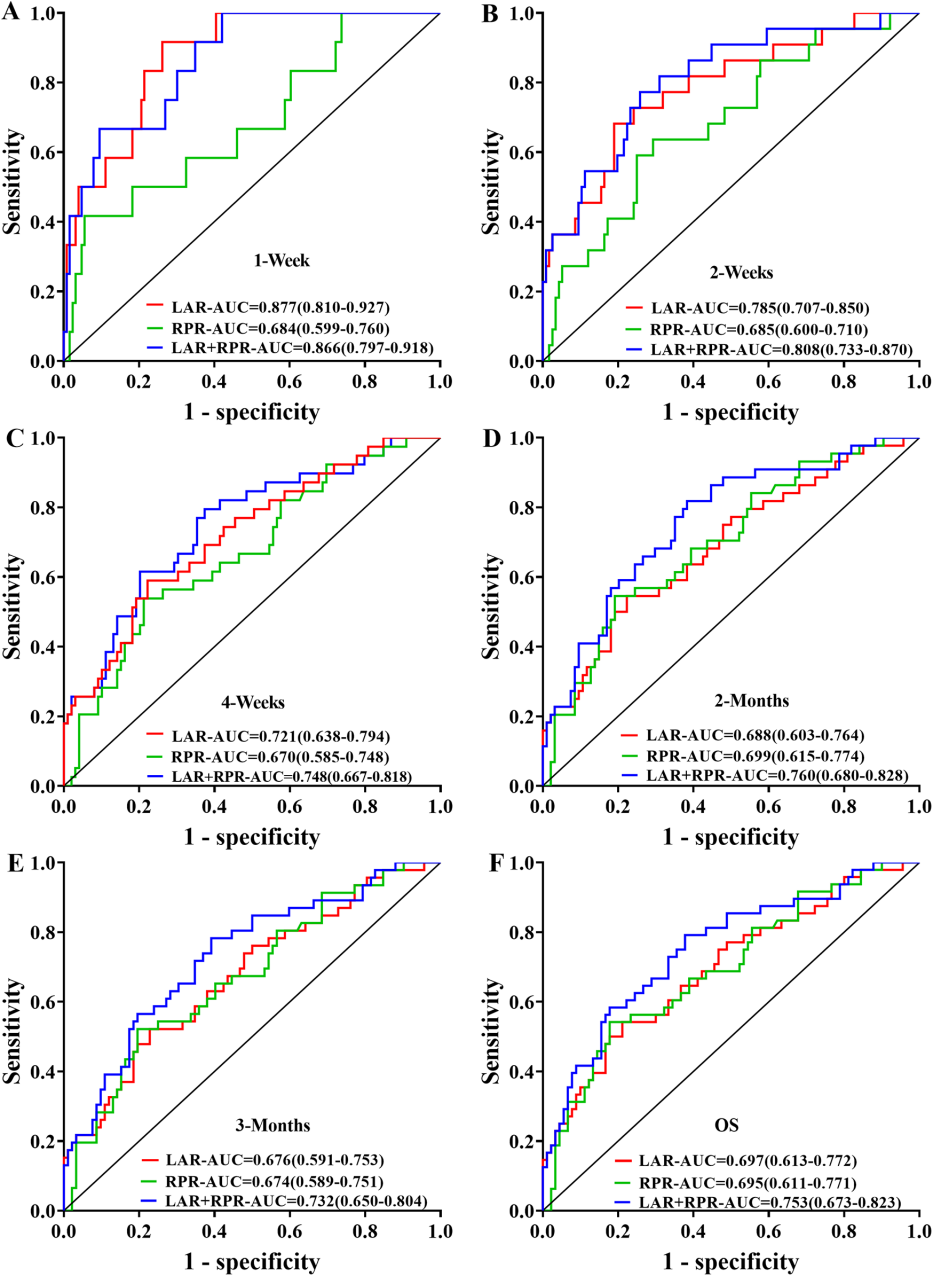
Receiver operating characteristic (ROC) curves were generated to compare the prognostic predictive performance of LAR, RPR, and their combination (LAR+RPR) across different time intervals: **(A)** 1 week, **(B)** 2 weeks, **(C)** 4 weeks, **(D)** 2 months, **(E)** 3 months, and **(F)** overall survival (OS). The curves illustrate the sensitivity versus 1-specificity relationships with corresponding area under the curve (AUC) values, showing the predictive capacity of each parameter at various clinical follow-up points.

To evaluate the prognostic significance of LAR and RPR for sHLH patients, we analyzed their predictive value across various time intervals (1 week, 2 weeks, 4 weeks, 2 months, and 3 months). LAR consistently demonstrated strong predictive performance, with AUC values exceeding 0.7 at 1 week, 2 weeks, and 4 weeks, and remaining above 0.6 at 2 and 3 months. Conversely, the predictive value of RPR remained stable across all time intervals, with AUC values ranging from 0.684 to 0.699. Comparative analysis of RPR, LAR, and their combined predictive ability (LAR-RPR) revealed that at week 1, both LAR and LAR-RPR outperformed RPR alone (*P* = 0.047 and *P* = 0.013, respectively), At week 2, LAR-RPR exhibited significantly superior performance compared to RPR alone (*P* = 0.032). No statistically significant differences were observed at other time points (*P* > 0.05). While the combined use of RPR and LAR did not significantly increase the predictive efficacy, both indices demonstrated commendable prognostic value (AUC > 0.6, *P* < 0.05). Notably, LAR showed significantly improved predictive performance in the early stage (≤4 weeks), with an AUC > 0.7 (*P* < 0.05) (Figure 5).

## 4 Discussion

Prognostic factors for HLH have been widely studied, revealing multiple independent risk factors associated with poor outcomes. These factors include laboratory, clinical, and dynamic variables (6, 20–25), which highlight the complexity of HLH prognosis, and underscore the importance of early risk stratification that integrates clinical, laboratory, and dynamic markers to guide treatment and improve prognosis in HLH patients. Previous studies have highlighted the prognostic value of blood inflammatory composite markers in inflammatory diseases, emphasizing their role in risk stratification. However, limited research has been conducted on the association between these markers and the prognosis of sHLH in children. In this retrospective analysis, we evaluated data from 138 pediatric patients with sHLH, aiming to assess the relationship between blood inflammatory composite markers and prognosis. The key findings include: (1) An overall mortality rate of 34.8% (48/138) among the cohort; (2) Significantly higher in-hospital mortality rates in patients with elevated LAR or RPR compared to those with lower LAR or RPR; and (3) High LAR or RPR levels during hospitalization showed preliminary prognostic relevance for adverse outcomes in pediatric sHLH, based on multivariate analysis within this proof-of-concept cohort.

Previous studies have underscored the prognostic relevance of RDW across various conditions, including cancer, sepsis, and inflammatory bowel disease (26–28). Recent studies on adult HLH have confirmed the correlation between elevated RDW and poor prognosis (29, 30). Similarly, our findings reveal a significantly elevation in RDW levels among deceased sHLH patients, with an RDW ≥ 14.35 showing potential as an independent prognostic indicator. RPR, an established marker of inflammatory processes, has also been recognized as a significant prognostic factor in various inflammatory diseases (12, 30–33). In studies on septic patients, higher RPR levels were associated with increased 28-day mortality rates (32). Similarly, retrospective analysis in adult HLH patients revealed a higher RPR in deceased patients, with RPR identified as an independent risk factor (30). Notably, a study of 179 adult HLH patients found that patients with an RPR > 0.33 exhibited a notably lower survival rates compared to those with RPR ≤ 0.33, with an AUC of 0.637 (95% CI 0.562–0.708) for RPR as a standalone prognostic predictor (33). Our study of 138 pediatric sHLH patients aligns with these findings, revealing significantly higher RPR level in the deceased group compared to survivors. Patients with RPR ≥ 0.36 exhibited significantly reduced survival rates. Importantly, even after adjusting for gender, age, etiology, and other laboratory parameters, RPR retained a statistically significant association with prognosis. These results suggest a potential association between RPR and outcomes in pediatric sHLH, supporting its exploratory value as a candidate marker for early prognostic assessment.

Previous studies have established a correlation between LDH, serum albumin, and HLH prognosis (34, 35). The combination of LDH and serum albumin, represented by the LAR, reflects both inflammation and nutritional status. LAR has been identified as a prognostic factor in various conditions, including sepsis, cancer, and inflammatory diseases (14, 36, 37). For instance, LAR has been linked with increased mortality risk at 28 and 90 days in sepsis patients and recognized as an independent risk factor for mortality (14). Another study identified LAR as an independent adverse prognostic factor in lower respiratory tract infections with a strong predictive value (AUC = 0.808) (36). Although no previous reports have explored the correlation between LAR and HLH prognosis, our findings reveal significantly higher LAR levels in the deceased group compared to survivors. This increase may be attributed to hypoalbuminemia caused by capillary leak associated with endothelial activation during inflammation process in sHLH. Additionally, patients with LAR ≥ 56.02 exhibited significantly reduced overall survival rates compared to those with LAR < 56.02, indicating that higher LAR levels may be associated with poorer prognosis. Multivariate Cox regression analysis further supported the potential of LAR as an independent prognostic marker in pediatric sHLH. These findings suggest that monitoring LAR values during HLH diagnosis and treatment may help in the early identification of critically ill patients, enabling timely intervention to potentially slow or prevent disease progression.

Previous studies have also highlighted the prognostic relevance of ALBI, De Ritis, and PNI in various inflammatory diseases (10, 11, 15, 38). In our study, we observed increased ALBI and De Ritis ratio levels and decreased PNI levels in the deceased group compared to survivors. While these factors showed an association with sHLH prognosis, they did not achieve statistical significance in multivariate analysis.

In summary, our exploratory analysis identified LAR and RPR as potential independent prognostic markers among eight routinely available inflammatory indices in pediatric sHLH. These factors represent simple, convenient, and cost-effective parameters derived from routine peripheral blood analysis. Our findings suggest that LAR and RPR may serve as potential biomarkers for indicating early and overall prognosis in pediatric sHLH. Notably, even after adjusting for confounding variables such as age, gender, etiology, and other inflammatory composite parameters, both markers retained independent associations with outcomes, supporting their possible utility for early risk stratification in this undifferentiated population. However, this study has certain limitations, including: (1) This analysis relied on static laboratory values at diagnosis without capturing dynamic changes over time, which may have reduced the sensitivity and timeliness of prognostic assessment. Longitudinal monitoring is needed to improve predictive accuracy and clinical utility. (2) As an initial proof-of-concept, these findings warrant further validation in larger multicenter cohorts with etiological stratification to clarify the broader prognostic utility of LAR and RPR in pediatric sHLH. (3) The potential influence of therapeutic strategies on dynamically measured variables introduces variability that may affect predictive accuracy. Differences in treatment approaches across medical centers further compound this limitation, potentially affecting the generalizability of the results. Future research should explore these aspects to enhance the understanding of LAR and RPR as prognostic markers.

## 5 Conclusion

In this proof-of-concept study of pediatric patients with sHLH, several routinely available composite inflammatory markers were found to be associated with overall survival. Specifically, elevated RPR (≥0.36) and LAR (≥56.02) demonstrated potential prognostic relevance and were identified as independent risk factors for poorer outcomes. However, their combined use did not significantly improve predictive performance over individual assessments. These findings suggest that RPR and LAR may serve as practical, accessible indicators to aid early risk stratification in pediatric sHLH, particularly in settings where advanced immunological tests are not readily available. Nonetheless, their predictive utility is not absolute and may vary with underlying etiology, disease heterogeneity, and treatment response. Therefore, these markers should be interpreted within the context of a comprehensive clinical assessment. Further validation in larger, multicenter, and etiologically stratified cohorts is necessary.

## Data Availability

The original contributions presented in the study are included in the article/Supplementary Material, further inquiries can be directed to the corresponding authors.
